# Comparing Single-Page, Multipage, and Conversational Digital Forms in Health Care: Usability Study

**DOI:** 10.2196/25787

**Published:** 2021-05-26

**Authors:** Aleeha Iftikhar, Raymond R Bond, Victoria McGilligan, Stephen J Leslie, Khaled Rjoob, Charles Knoery, Ciara Quigg, Ryan Campbell, Kyle Boyd, Anne McShane, Aaron Peace

**Affiliations:** 1 Computing Engineering and Build Environment Ulster University Jordanstown United Kingdom; 2 Centre for Personalised Medicine Ulster University Londonderry United Kingdom; 3 Cardiac Unit Raigmore Hospital Inverness United Kingdom; 4 Department of Cardiology Altnagelvin Hospital Western Health and Social Care Trust Londonderry United Kingdom; 5 Faculty of Arts, Humanities & Social Sciences Ulster University Belfast United Kingdom; 6 Letterkenny University Hospital Letterkenny Ireland

**Keywords:** digital forms, health care, usability evaluation, single-page form, multipage form, conversational forms

## Abstract

**Background:**

Even in the era of digital technology, several hospitals still rely on paper-based forms for data entry for patient admission, triage, drug prescriptions, and procedures. Paper-based forms can be quick and convenient to complete but often at the expense of data quality, completeness, sustainability, and automated data analytics. Digital forms can improve data quality by assisting the user when deciding on the appropriate response to certain data inputs (eg, classifying symptoms). Greater data quality via digital form completion not only helps with auditing, service improvement, and patient record keeping but also helps with novel data science and machine learning research. Although digital forms are becoming more prevalent in health care, there is a lack of empirical best practices and guidelines for their design. The study-based hospital had a definite plan to abolish the paper form; hence, it was not necessary to compare the digital forms with the paper form.

**Objective:**

This study aims to assess the usability of three different interactive forms: a single-page digital form (in which all data input is required on one web page), a multipage digital form, and a conversational digital form (a chatbot).

**Methods:**

The three digital forms were developed as candidates to replace the current paper-based form used to record patient referrals to an interventional cardiology department (Cath-Lab) at Altnagelvin Hospital. We recorded usability data in a counterbalanced usability test (60 usability tests: 20 subjects×3 form usability tests). The usability data included task completion times, System Usability Scale (SUS) scores, User Experience Questionnaire data, and data from a postexperiment questionnaire.

**Results:**

We found that the single-page form outperformed the other two digital forms in almost all usability metrics. The mean SUS score for the single-page form was 76 (SD 15.8; *P*=.01) when compared with the multipage form, which had a mean score of 67 (SD 17), and the conversational form attained the lowest scores in usability testing and was the least preferred choice of users, with a mean score of 57 (SD 24). An SUS score of >68 was considered above average. The single-page form achieved the least task completion time compared with the other two digital form styles.

**Conclusions:**

In conclusion, the digital single-page form outperformed the other two forms in almost all usability metrics; it had the least task completion time compared with those of the other two digital forms. Moreover, on answering the open-ended question from the final customized postexperiment questionnaire, the single-page form was the preferred choice.

## Introduction

### Background

**Figure 1:**
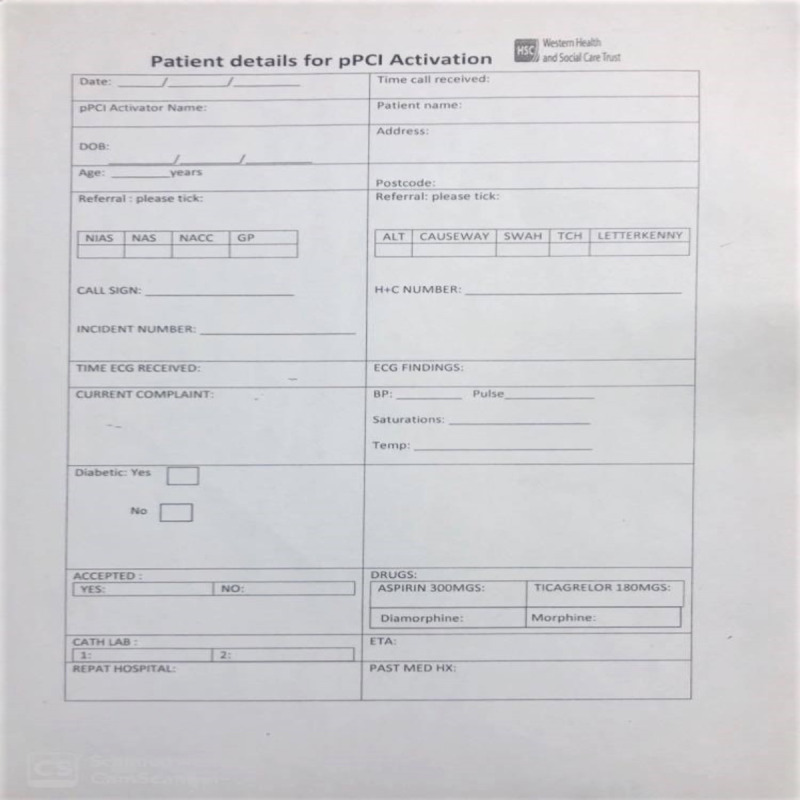
The current paper-based form being used at Altnagelvin Hospital.

Currently, when a primary percutaneous coronary intervention (PPCI) referral is made, the nurse activator in the coronary care unit will triage the patient using written notes. Typically, when a patient experiences chest pain, paramedics arrive and record an electrocardiogram. If the paramedic suspects a heart attack, they will then contact the PPCI department at a hospital and describe the symptoms and electrocardiogram findings to an activator nurse, who then completes a paper form shown in Figure 1 and decides whether patients need to be accepted or turned down.

This is not unusual, as most hospitals and cardiac care units often rely on paper-based forms for data entry for patient admission or drug prescriptions and other general procedures. Working with paper-based systems can be challenging, especially when a health care staff works in a sensitive and highly stressful environment, such as cardiac care. Digitalization is slowly being introduced into the health service to improve the medical workflow at different stages and levels. Many applications serve many purposes, including facilitating communication between a patient and a provider, remotely monitoring patients, and measuring population health objectives, such as disease trends. The collected information can be used to make informed decisions about health care services, either at the population level or individual level, to improve care [1]. Electronic health record (EHR) adoption rates have introduced efficiencies in health care operations, such as instant access to information, improved practice management, and reduced paperwork. Other findings relate to the impacts that EHR systems have on physicians’ time, expertise, and learning. The literature also present findings on the impact of EHR systems at the length (and sometimes the accuracy) of the clinical notes [1]. Again, multiple factors contribute to these intrusions, including computer availability, physical positioning of computers, design of the user interface, length of the forms, and procedure of filling the forms. Physician-residents have to use EHR systems because of their mandatory nature; however, if they had a choice or power, most physicians would likely use the paper chart [1]. Recent work has suggested that clinical decision support systems integrated within EHR systems hold the promise of improving health care quality. To date, the effectiveness of clinical decision support systems has been less than expected, especially concerning the ambulatory management of chronic diseases [2]. Nevertheless, although digitization is a drive to improve services, clinicians may not always welcome new digital systems [3]. Certain hurdles may make them reluctant to adopt a digital system, such as prior investment and familiarity with a current system (known as *baby duck syndrome*) [4] and availability, training, and the position of the system [3]. Although it is feasible to use digital forms in medicine, it has its design constraints, including limited display size and the challenge of replicating the user experience of paper forms or checklists [5]. These constraints can be handled; however, there are many conflicting guidelines available on appropriate user-centric designs. Bevan [6] analyzed usability guidelines to inform a user-centric design. Bevan [6] compared these usability methods with those found in textbooks and discussed the most effective way to present user-centric guidelines through a website.

### Prior Work

Similar to other fields, digitalization and digital transformation play an essential role in health care. Health care technologies are rapidly growing and evolving; for example, EHR systems are becoming routine [7]. Moreover, different digital forms are being used in medicine in several ways, such as recording triage or referral data, observations of vital signs, and synoptic reporting in pathology. Digital forms and digital checklist systems are computer-based instructions for recording or performing actions as part of managing tasks [6]. Numerous research studies have studied digital forms in medicine, especially the use of mobile digital forms to support high-quality data collection [8]. It has been stated that electronic reporting is often more efficient and representative with higher rates of data completions [9] and is more effective for supporting clinical decision making. One study stated that using a standard single-page digital form called the standardized outpatient osteopathic note form was more efficient and accurate than the paper-based equivalent [10]. There has been a recent demand for smart checklists (often digital) in medical procedures to reduce iatrogenic or medical errors [11]. A comparison of team performance used a paper checklist with a digital checklist to determine whether digitizing a checklist led to improvements in task completion. The researchers found some improvements in team performance when using the digital checklist [12]. A study developed and evaluated two different versions of a tablet-based cognitive aid to support in-hospital resuscitation team leaders. They suggested that digital cognitive aids may help increase effectiveness and eventually improve patient safety [13]. Chatbots and conversational forms are also being tested in different fields. A comparison of surveys presented as traditional web pages versus chatbot or conversational style surveys (text-based virtual agent) found that participants who used the chatbot style survey produced higher-quality data [14].

### Goal

Given the demand for effective digital forms, there is a need to research and discover the best-practice interaction design guidelines for designing digital health forms. In this study, we designed three different digital form styles to replace a paper form that is used for patient referrals to a PPCI service. To contribute to future digital form design guidelines in health care, the study also aims to compare the usability of all three forms to analyze which form styles work best for health care professionals. However, measuring usability is difficult because usability does not refer to a single property; rather, it combines several attributes [15]. According to the standard International Organization for Standardization 9421-11, usability is the effectiveness, efficiency, and satisfaction by which users must achieve a certain goal in a particular environment [16]. This study aims to measure and compare the usability of these three interactive form designs in a counterbalanced experiment in a controlled laboratory at Altnagelvin Hospital.

## Methods

### Overview

Textbox 1 shows the adopted structure describing the usability test flow for this study.

**Adopted structure describing the usability test flow for this study.**

**Objective**

- The focus or aim is to compare different digital form designs to evaluate which digital form has greater usability.

**Participants**

- The total study population consisted of 20 health care staff who were either cardiac nurses or research nurses.

**Apparatus**

- Microsoft surface pro to display the digital forms and to facilitate user interaction, a microphone to record the user’s think-aloud data, and screencasting software to video record the user interactions with the digital forms
- Questionnaires (System Usability Scale and User Experience Questionnaire) to measure usability and R-studio for data analysis

**Outcomes**

- System Usability Scale usability score, usability errors, and task completion times

**Procedure**

- Counterbalanced experiment to avoid any learning bias
- Typical patient scenarios were presented to the user to facilitate the form completions.

**Data analysis**

- Summary analysis of System Usability Scale scores, User Experience Questionnaire results, task completion times, error rates using descriptive statistics, and boxplots
- Hypothesis testing (*t* tests, where α<.05) was used to determine statistical significance between System Usability Scale scores and task completion times

### Data Set

This study involved the analysis and comparison of three different digital form designs that were developed as candidates for recording patient referrals to a PPCI service at Altnagelvin Hospital (Northern Ireland, the United Kingdom). This study only aims to compare the digital forms, as there are already studies that compare paper forms with digital or electronic forms [17-22]. The paper form was only included to compare the task completion time, and no other metrics were recorded to measure the usability of the paper form. The total study population consisted of 20 health care staff (men: 4/20, 25%; age: 30-39 years) who were either cardiac nurse activators or research nurses. This study included 10 cardiac nurse activators and 10 research nurses.

### Development of Digital Forms

**Figure 2:**
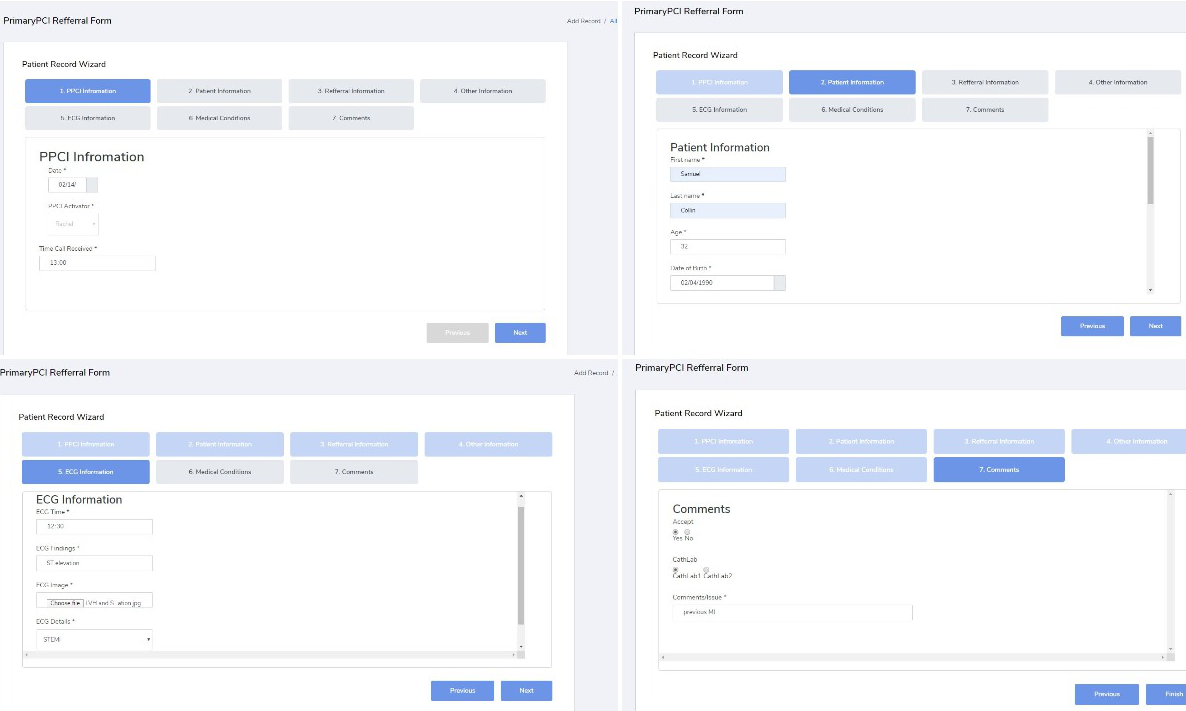
Screenshots of a part of the single-page form.

**Figure 3:**
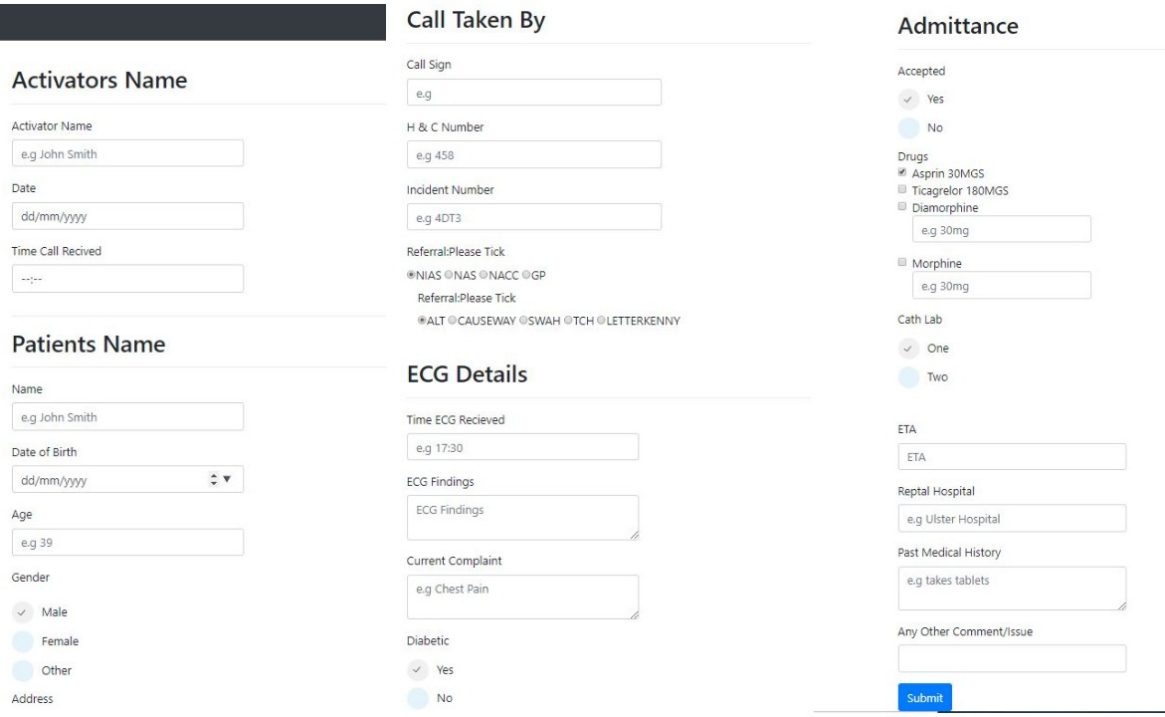
Screenshots of the screens from the multipage form.

**Figure 4:**
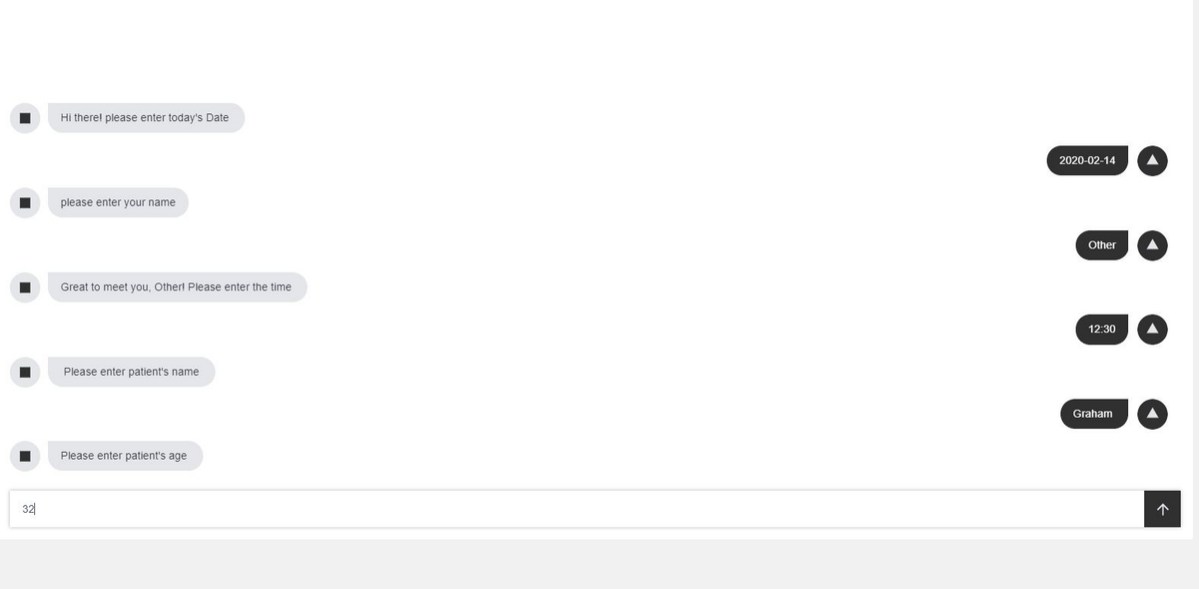
Screenshots of the conversational form.

The three different digital forms were developed using the HTML 5 and cascading stylesheets (CSS3) following the model view controller paradigm. An open-source scripting library was used to convert the web form into a conversational form [23]. The three digital form designs included (1) a single-page form, (2) a multipage form, and (3) a conversational form (chatbot), as shown in Figure 2, Figure 3, and Figure 4, respectively. The single-page form is where all the input fields are organized and given on a single screen, whereas the multipage form segments the input fields over seven different screens or pages in the form of tabs. In this case, the user completes one page of the form and then navigates to the next tab or section. In the conversational form, the questions are presented to the user in a preset sequence of questions where the user can type in the answer or choose from a series of options. The rationale and expected pros and cons of each type of digital form are presented in Table 1.

**Table 1:** Expected pros and cons of the three digital forms.

Form type	Pros	Cons
Single-page form	Easy to understand Common form style and meets expectations User can view all questions and input fields expected of them User can predict the work required to complete the form Easy to navigate to all information on a single page	High information rate. Busy looking screen with possible clutter User can be distracted by the number of questions required The screen can require more mental workload to interpret Information overloading can result in visual hierarchy issues
Multipage form	Deconstructing a task into subtasks reduces cognitive load Less distracting for users User can be guided and focused on a small set of related questions Creates a sense of progression	Additional interactions (clicks) to navigate to the different sections Misleads the user into thinking the form is shorter than it is It might take longer to complete User needs to navigate to change answers from a previous form subsection
Chatbot form	Easy to use Fewer distractions given only one question is presented per interaction It is akin to everyday human interaction or to being interviewed and hence engenders focus Less cognitive demand It is novel	Not a common form style Editing previous input could be cumbersome and require a lot of interactions It seems too playful for formal settings such as medicine Preset sequence to follow

### Usability Testing Protocol

The participants identified to be suitable and interested in participating were given a participant information sheet, and written informed consent was obtained from all participants interested in the study (by the author).

**Figure 5:**
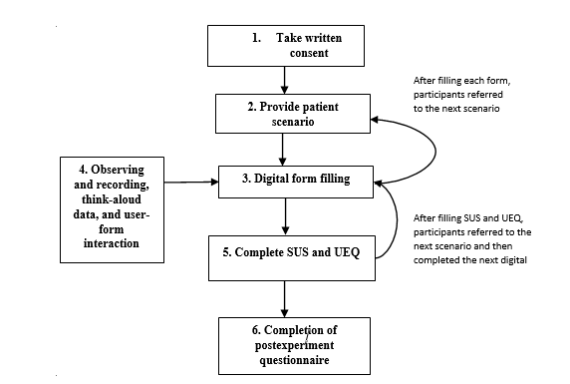
Flowchart depicts the usability testing session flow. SUS: System Usability Scale; UEQ: User Experience Questionnaire.

This study tested three different digital forms in a simulated setting where each participant was given a brief tutorial on how to use the tablet PC (Microsoft Surface Pro) that hosted the digital forms. Each participant was provided with the same four PPCI triage–simulated scenarios written on a sheet as shown in Multimedia Appendix 1 and was asked to complete a paper form (standard routine clinical form) and each of the three digital form designs. The sequence of when the subject interacted with the digital forms was counterbalanced to avoid any learning bias. Each session took approximately 60 minutes for each participant. Figure 5 shows the session protocol.

The researcher observed the participants while they completed the forms, and notes were taken to record usability issues. Form completion was recorded using a screen recording software (FreeScreenRecorder by Screencast-O-Matic [24]) on the tablet. Usability factors were evaluated, including user satisfaction; error rate (error rate was noted while observing the participants filling in the form as well as after the session by watching the recorded video); classification of the severity of the usability issues or error analysis, which was recorded using Nielsen’s 4-star severity scale, that is, cosmetic to severe (1-4) [25]; task completion time (each form completion time was noted for each participant using a stopwatch and cross-checked with the video timings); and ease of use (ease of use is a basic concept that describes how easily users can use a product). All questionnaires had questions related to ease of use. Moreover, the error rate and task time also depict the user’s ease of using a particular form design. After completing each form, participants were asked to complete the System Usability Scale (SUS) questionnaire [26].

The SUS is commonly used and is a validated questionnaire consisting of 10 items. The scoring of this questionnaire provides a usability score ranging from 0 to 100. An SUS score of >68 is considered above average, and anything <68 is considered below average. A study by Tullis and Stetson [27] performed a comparison of questionnaires for assessing website usability using the Computer System Usability Questionnaire [28]. Brooke [29] developed the SUS in 1996 [29]. The SUS uses a 5-point scale, ranging from strongly agree to strongly disagree. According to Bangor et al [30], the SUS is flexible in assessing a wide range of technologies. The SUS is also relatively quick and easy to use by study participants. Additionally, the SUS provides a single score on a scale that is easily understood. User experience was also recorded using the standard User Experience Questionnaire (UEQ). The UEQ measures six factors: attractiveness, perspicuity, efficiency, dependability, stimulation, and novelty [31]. This questionnaire can be used in different scenarios to record the user experience [32]. The UEQ provides the user with a bidirectional Likert scale with both positive and negative aspects of the system for them to rate, such as questions with positive connotations (easy to learn and creative) and questions with negative connotations (annoying, boring, and not interesting). The questionnaires were completed for all three forms to benchmark and compare the usability of the user interfaces for both positive and negative attributes of each form [33].

A customized postexperiment questionnaire was administered at the end of the session. The postexperiment questionnaire was a final customized researcher-created questionnaire. This questionnaire had 21 usability-related questions that focused more on the needs and types of preferred forms and preferred features.

The recorded data were then analyzed to compare the usability and user experience for each form. This process was used for each subject and also consisted of (1) the concurrent think-aloud protocol and a brief interview, (2) screen recording of the user interactions, and (3) usability evaluation of the final digital form prototypes (60 usability tests: 20 subjects×3 forms). Each participant was observed while they completed each digital form. The screencast was used to analyze and evaluate the user’s behavior.

The data were collected through observations made while the participants were interacting with the digital forms. We then computed the error rate, task completion time, and user satisfaction. For the error rate analysis, a possible error list was made for each form design, and then, the number of errors was noted for each digital form against each user. The least task completion time for a form and the lowest error rate for a particular form can indicate the best form eliciting the highest user satisfaction. User satisfaction was also more explicitly covered in the SUS and UEQ. The postexperimental questionnaire also asked the user about their preferred choice of digital form design.

### Data Analysis

Different statistical metrics are used, including median, mean, and SD for the variance. The paired two-tailed *t* test was used to compare any differences between the task or form completion times and the SUS scores between all the three forms. Owing to the multiple statistical tests on the same data sets, Bonferroni corrections were used. Pearson correlation was used to identify any association between the SUS scores and the task completion times. It was not feasible to perform correlation analysis between other usability factors, such as UEQ answers and error rates, given that they generate categorical results, unlike SUS and the task time, which are numeric values.

### Ethical Aspects

Research governance permission was granted by the Western Health and Social Care Trust (WT 19/08, Integrated Research Application System 262557) and complied with the Declaration of International Research Integrity Association (Multimedia Appendix 2).

## Results

### SUS Score Analysis

**Figure 6:**
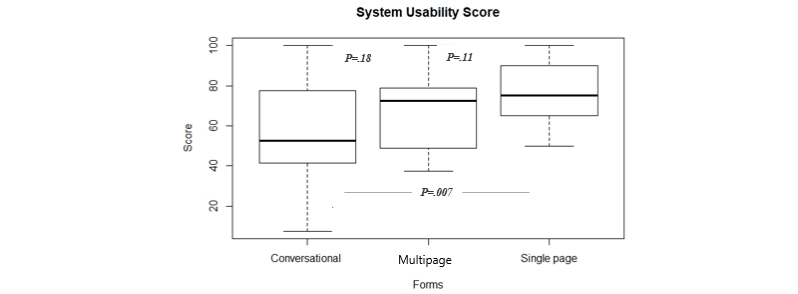
Boxplot for the average System Usability Scale score of each form. The single page had a mean System Usability Scale score of 76 (SD 15) and outperformed the usability of the multipage and conversational forms with mean System Usability Scale scores of 67 (SD 17) and 57 (SD 24), respectively. Even with a β coefficient of .015, the results are still significant.

On the basis of the research, an SUS score of >68 is considered above average [34]. With a mean SUS score of 76 (SD 15), the single-page form outperformed the usability of the multipage and conversational forms. The multipage form was on the borderline with a mean score of 67 (SD 17). The conversational form attained the least scores in the usability testing and it was the least choice of users, with a mean score of 57 (SD 24). The *t* test indicated statistical significance between the conversational and single-page forms. Figure 6 shows a boxplot of the SUS scores for each digital form. Even with the Bonferroni-corrected α value (.015), the results were still statistically significant.

### UEQ Interpretation

**Figure 7:**
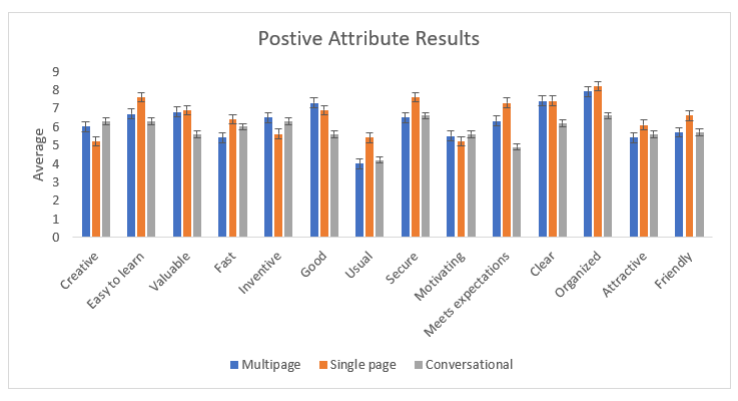
Bar chart showing positive attribute results of the User Experience Questionnaire for all three forms. The single-page form has higher averages for the positive attributes than those of the other two digital forms.

**Figure 8:**
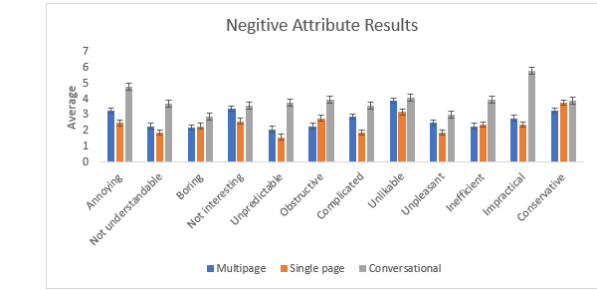
Bar chart showing negative attribute results of the User Experience Questionnaire for all three forms. The conversational form had higher averages for the negative attributes than those of the other two forms, which suggests that the conversational form had the least usability.

The UEQ used in this study was modified from the original version by making it unidirectional and also included the one-sided factors. The single-page form mostly had higher averages for the positive attributes than the other two digital forms. The conversational form scored higher averages in the negative attributes, which suggests that the conversational form had the least usability. Figures 7 and 8 show the mean average ratings for each UEQ question for each digital form.

### Task Time or Form Completion

**Figure 9:**
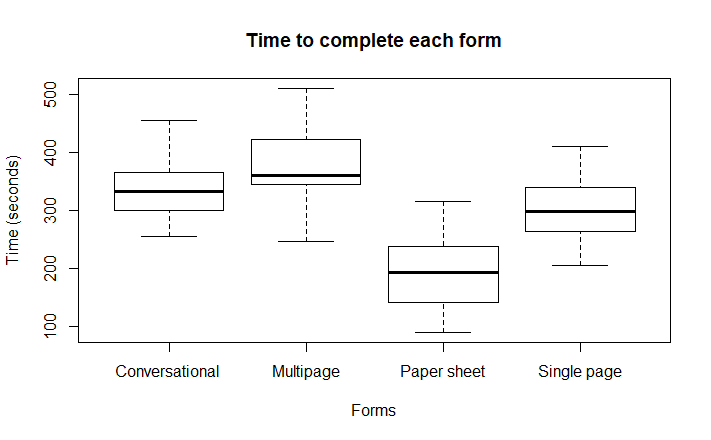
Boxplot for the average form completion time of each form. The primary percutaneous coronary intervention activator nurses took the least time for the paper form, as they are currently using this for primary percutaneous coronary intervention referrals. However, the research nurses who had no prior exposure to this paper form took almost as much time as the time activator nurses took to complete the digital forms (mean 224 seconds, SD 54 seconds vs mean 298, SD 60 seconds; *P*<.001).

Task completion refers to the total time a user takes to complete each form. Participants took the least time to complete the paper form. However, the least mean time was recorded for the single-page form, followed by the conversational form among the three digital forms. Users took longer to complete the multipage form. Figure 9 shows a boxplot of task completion times for each form. The PPCI activator nurses took the least time for the paper form, as they are currently using this for PPCI referrals. However, the research nurses who had no prior exposure to this paper form took almost as long as they took to complete the digital forms (mean 224, SD 54 seconds vs mean 298, SD 60 seconds; *P*=.001).

**Figure 10:**
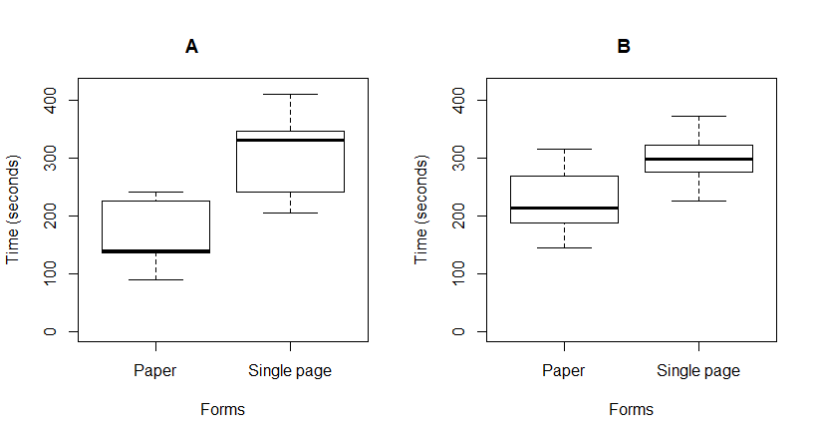
Boxplot for the average form completion time of activators versus research nurses. (A) Activator nurses’ form completion time and (B) research nurses’ form completion time.

On the other hand, the activator nurses who took the least time to complete the paper form took almost twice the amount of time to complete the digital form compared with the paper form (165, SD 55 s vs 301, SD 68 s; *P*<.001). The boxplot in Figure 10 shows the mean time of both groups to complete the paper and digital forms. The paired *t* test is shown in Table 2, where the single-page form shows significance (*P*<.001) with the multipage form and paper form. The multipage form and the conversational form task completion times showed significance (*P*<.001) with the paper form only.

**Table 2:** *P* values between the completion time of all forms.

Form comparisons	*P* value
Single-page form and multipage form	<.001
Single-page form and conversational form	.02
Single-page form and paper sheet	<.001
Multipage form and conversational form	.10
Multipage form and paper sheet	<.001
Conversational form and paper sheet	<.001

### Correlation: SUS Score and Task Time

**Figure 11:**
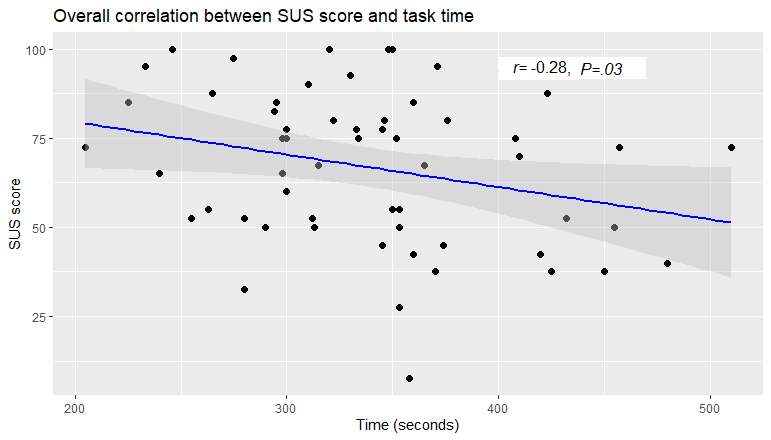
Scatterplot for the overall correlation between the System Usability Scale score and task completion time. There was a weak correlation (*r*=−0.28) between the System Usability Scale score and form completion times. This shows that the task completion time alone does not measure the usability of a system. SUS: System Usability Scale.

**Figure 12:**
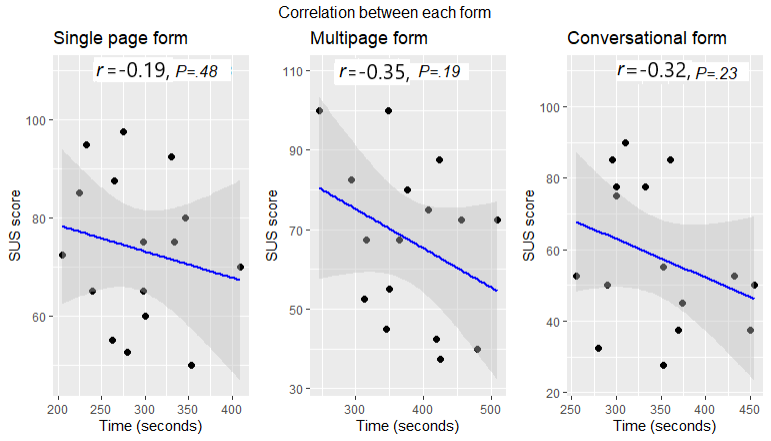
Scatterplot for the overall correlation between the System Usability Scale score and each form's completion time. SUS: System Usability Scale.

There was a weak correlation (*r*=−0.28) between the SUS score and form completion time (Figure 11). This shows that task completion time alone does not measure the usability of a system. Figure 12 shows the scatterplot for the overall correlation between the SUS score and each form completion time.

### Error Rate and Classification

**Figure 13:**
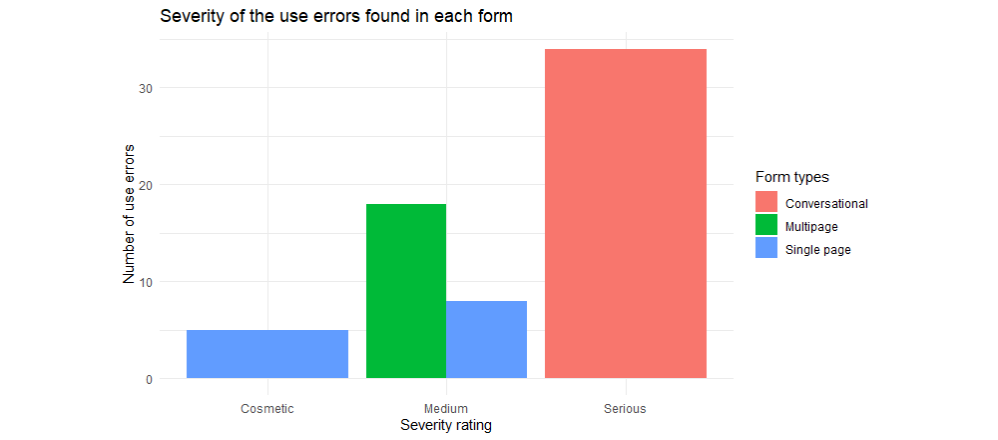
Bar graph for each form’s error severity. The multipage form errors were 69% medium errors, whereas the single-page form had only 31% medium errors and very few cosmetic errors.

Upon inspection of the video screen recordings, the use errors and their frequency were recorded. A use error can have 1 of 4 severity ratings according to Neilson’s 4-star severity scale, that is, cosmetic, medium, serious, or critical. There were no critical use errors; however, there were many serious use errors in the conversational form. The multipage form errors were 69% medium errors, whereas the single-page form had only 31% medium errors and very few cosmetic errors. Figure 13 shows a bar graph of the error severity of each form.

On the basis of this usability study, approximately 83 use errors (average severity 3.0) were discovered in the conversational form, 35 use errors (average severity 2.0, SD 0) were discovered in the multipage form, and 21 use errors (average severity 1.76, SD 0.44) were discovered in the single-page form. The severity of these use errors is shown in Figure 13.

### Postexperiment Questionnaire

Approximately half of the participants preferred the single-page form. In response to an open-ended question, the users mentioned that the single page was “easy to complete,” “easy to understand,” “well-marked and separated,” and “clearer” and that “all the information is available to see at once.” For the multipage form, the users said the “entire information isn’t available” and that they “don’t like to navigate.” For the conversational form, the users said that it was “unpredictable” and “difficult to understand and use” and that they “couldn’t go back easily to the options if they need to or want to.”

## Discussion

### Principal Findings

This study has shown that a single-page digital form outperformed the multipage and conversational forms while performing usability evaluation for the three digital forms designed for PPCI referrals to better understand the usability needs of nurses. This is an interesting finding, as the conversational form was previously used successfully to aid in different areas [35,36]. In terms of task completion times, the single-page form achieved the minimum completion time, followed by the conversational form.

The correlation analysis between the SUS score and task time showed no strong relationship, indicating that task completion time alone cannot measure the usability of a system. All the standard usability metrics considered in this research concluded that the single-page digital form performed better than the multipage and conversational forms. Moreover, while answering an open-ended question in the final questionnaire, more than half of the participants chose the single-page form as their preferred choice. Some of the reasons for preferring the single-page form were that it is easy to complete, easy to understand, well-marked and separated, clear, and all the information is available to see on one screen. For the multipage form, participants did not seem to like navigating between the pages. For the conversational form, participants found it more unpredictable; difficult to understand and use; and, most importantly, to be unable to conveniently go back to change data inputted if they needed to.

Usability assessment and appropriate form design or form design guidelines are vital for health care departments. For form filling in health care, if the form is not well designed, people will have to think harder to complete it. If they think harder, it means they will take longer to fill in the form, so they could miss information or skip it or even enter wrong information. If people take long time to fill the forms, it takes them away from the actual patient care. If they make mistakes and put in wrong information, any algorithms, data analysis, or dashboards that use those data would be wrong. Clinical strategies and decision making at the board level or hospital level based on those data would be wrong because a nurse had not completed a digital form properly. The fact that the digital form is being used routinely and at a high frequency makes their usability crucial because you will think that a system as simple as a form should not require a high mental workload. It should be as intuitive and as simple as possible. A digital form impacts algorithm development and policy decision making because much of the data are based on policy decision making, which means that if data are wrong, then the policies are also wrong. If people are not putting in the right data, then policy decisions will be faulty as well. In this day and age, we make many decisions based on the data, so data can be either new oil or a new snake oil if the data are misleading or wrong. Data are substantial if it is correct, but it can lead to bad decisions if data are not correct. The results from the study clearly show that a single page from has better usability overall than its multipage and conversational form counterparts. This has implications for form design moving forward but, in many ways, reinforces good user experience design guidelines when it comes to form design [37]. By using single-page forms, they allow the layout to be simplified and make a form easily scannable. When people first see a form, they will perceive how long it will take for them to complete it by scanning the form. Therefore, perception does play a role. The more complex it looks, the more likely people will abandon the process. There is also the interaction cost or the *reservoir of goodwill*. Filling in web forms represents a sum of effort both cognitively and physically that people must put in when interacting with a web form to reach a goal. The more effort required, the less usable the form is. The reservoir of goodwill diminishes, and people abandon the process; single-page forms allow long forms to appear smaller by minimizing the number of fields that are seen at the same time. This creates the perception that the form is shorter than it really is. This is done via progressive disclosure, showing just what the people need on the screen at the right time. By also *chunking* breaking the form into steps allows people to process, understand, and complete information in a small portion at a time. The trend for web forms is this approach with web builders, such as Google forms [38] and typeform [39], using this approach.

### Limitations

The digital forms were trialed at only one hospital with a small group of health care professionals, and the usability results may differ at other centers. However, the ethical approval board is in the process of including another hospital site in the study to increase the number of participants. The study was conducted in a simulated scenario in which the location and patient presentation were simulated. Perhaps in real scenarios, participants would be under more pressure (eg, time pressure). Usability data were not recorded for the paper version. No usability data are available for the paper form, as the usability questionnaires (SUS and UEQ) are designed to assess digital interfaces. Paper forms are what health care staff are very familiar with and might bias any comparisons made. For example, they have already adopted paper systems and have become experts in paper form filling. Hence, it can be argued that it is unfair to compare paper form completion with digital form completion because this compares expert use with novice use. Moreover, another key limitation is that perhaps single-page digital forms are preferred because that format is also widely used and users might have already become familiar with these form styles.

### Future Work

How will people complete digital forms in the future? This is an interesting question, especially in the era of artificial intelligence. Perhaps there will be more intelligent smart speakers that will be used for completing forms, for example, an artificial intelligence algorithm that listens to the patient’s details and completes the form using natural language understanding. However, talking to a computer requires more effort than selecting options in a form. Further research is required to explore these ideas.

### Conclusions

In conclusion, the digital single-page form outperformed the other two forms in almost all usability metrics. The mean SUS score for a single page was 76 (SD 15), with the least task completion time when compared with the other two digital forms. Moreover, on answering the open-ended question, the single-page form was also the preferred choice. However, this preference might change over time as multipage and conversational forms become more common. For example, the conversational form’s SUS scores achieved a greater variance, indicating a possible dichotomy among participants regarding the perceived usability and usefulness of chatbot style form.

## Appendix

Multimedia Appendix 1 Simulated patient scenarios provided for form filling.

Multimedia Appendix 2 Ethical approval certificate/letter.

## Abbreviations

EHR: electronic health record
PPCI: primary percutaneous coronary intervention
SUS: System Usability Scale
UEQ: User Experience Questionnaire
