# Supplementary material for: Comparing Single-Page, Multipage, and Conversational Digital Forms in Health Care: Usability Study
Source: JMIR Hum Factors. 2021 May 26;8(2):e25787. doi: 10.2196/25787 (PMC8190652; doi:10.2196/25787)
Supplement: Multimedia Appendix 1 [file humanfactors_v8i2e25787_app1.pdf]

Appendix A

**Scenario 1:**

"A patient called Sarah Jones has just been referred at 11.00am, she has a sharp chest pain. Her age is 59 and she has been suspected with a heart attack. She was picked by the local ambulance service Londonderry and has been referred by NAS. She is expected to arrive Altnagelvin Hospital in 30minutes. She has diabetic and heart problem. She has already been treated with 30mg of Aspirin and 10mg Morphine. You are receiving this information from the local ambulance service. You have also received the ECG for the same patient at 11:05am suggesting ST elevation. This is incident number 25. Now you are required to fill the referral form for this same patient. The patients HC number is A2345678 and DOB is 24-08-1978. The pick up post code is BT37 0Gh. The observations are Bp 112/80, pulse 59, saturation 98 and temperature is 36.2."

**Scenario 2:**

"A patient called James Mathew has just been referred at 05.00pm, he is complaining chest pain. His age is 45 and he has been suspected with a heart attack. He was picked by the local ambulance service Londonderry and has been referred by NACC. He doesn't have any past medical history. He is expected to arrive Altnagelvin Hospital in 40minutes. He has already been treated with 20mg of Aspirin and 50mg ticagrelor. You are receiving this information from the local ambulance service. You have also received the ECG for the same patient at 04:55pm suggesting hyperacute T waves. This is incident number 05. Now you are required to fill the referral form for this same patient. The patients HC number is A87521468 and DOB is 12-10-1970. The pick up post code is BT40 0PP. The observations are Bp 125/70, pulse 69, saturation 108 and temperature is 36.0."

**Scenario 3:**

"A patient called Lisa Graham has just been referred at 1.00am, she is complaining chest pain. Her age is 39 and she has been suspected with a heart attack. She was picked by the local ambulance service Causeway and has been referred by NIAS. She has history of hypertension. She is expected to arrive Altnagelvin Hospital in 20minutes.She has already been treated with 20mg of Aspirin and 15mg Morphine. You are receiving this information from the local ambulance service. You have also received the ECG for the same patient at 01:00am suggesting ant ST elevation. This is incident number 27. Now you are required to fill the referral form for this same patient. The patients HC number is I2574258 and DOB is 28-04-1980. The pick up post code is GT25 0HRh. The observations are Bp 102/80, pulse 70, saturation 98 and temperature is 33.2."

**Scenario 4:**

"A patient called Samuel Collin has just been referred at 02.00pm, he is complaining chest pain. His age is 55 and he has been suspected with a heart attack. He was picked by the local ambulance service Letterkenny and has been referred by NACC. He has history of hypertension and diabetes. He is expected to arrive Altnagelvin Hospital in 40minutes. He has already been treated with 20mg of Aspirin and 50mg ticagrelor. You are receiving this information from the local ambulance service. You

have also received the ECG for the same patient at 01:58pm suggesting ST elevation. This is incident number 35. Now you are required to fill the referral form for this same patient. The patients HC number is H4569878 and DOB is 08-10-1970. The pick up post code is BT37 0PP. The observations are Bp 110/95, pulse 75, saturation 98 and temperature is 36.2.”
