# Supplementary material for: Comparing Single-Page, Multipage, and Conversational Digital Forms in Health Care: Usability Study
Source: JMIR Hum Factors. 2021 May 26;8(2):e25787. doi: 10.2196/25787 (PMC8190652; doi:10.2196/25787)
Supplement: Multimedia Appendix 2 [file humanfactors_v8i2e25787_app2.pdf]

London - Brighton & Sussex Research Ethics Committee
Health Research Authority
Ground Floor, Skipton House
80 London Road
London
SE1 6LH

Telephone: 02071048129

20 December 2018

Dr Victoria McGilligan
Centre for Personalised Medicine
CTRIC Building, Altnalgelvin Hospital
Ulster University, Londonderry
BT47 6SB

Dear Dr McGilligan

**Study title:**	**Feasibility study to develop a tool to improve clinical decision-making in the primary percutaneous intervention (pPCI) pathway**
**REC reference:**	**18/LO/2203**
**Protocol number:**	**n/a**
**IRAS project ID:**	**251710**

The Proportionate Review Sub-committee of the London - Brighton & Sussex Research Ethics Committee reviewed the above application in correspondence.

We plan to publish your research summary wording for the above study on the HRA website, together with your contact details. Publication will be no earlier than three months from the date of this favourable opinion letter. The expectation is that this information will be published for all studies that receive an ethical opinion but should you wish to provide a substitute contact point, wish to make a request to defer, or require further information, please contact hra.studyregistration@nhs.net outlining the reasons for your request. Under very limited circumstances (e.g. for student research which has received an unfavourable opinion), it may be possible to grant an exemption to the publication of the study.

Ethical opinion

On behalf of the Committee, the sub-committee gave a favourable ethical opinion of the above research on the basis described in the application form, protocol and supporting documentation, subject to the conditions specified below.

Conditions of the favourable opinion

The REC favourable opinion is subject to the following conditions being met prior to the start of the study.

Management permission must be obtained from each host organisation prior to the start of the study at the site concerned.

Management permission should be sought from all NHS organisations involved in the study in accordance with NHS research governance arrangements. Each NHS organisation must confirm through the signing of agreements and/or other documents that it has given permission for the research to proceed (except where explicitly specified otherwise).

Guidance on applying for HRA and HCRW Approval (England and Wales)/ NHS permission for research is available in the Integrated Research Application System, www.hra.nhs.uk or at http://www.rdforum.nhs.uk.

Where a NHS organisation's role in the study is limited to identifying and referring potential participants to research sites ("participant identification centre"), guidance should be sought from the R&D office on the information it requires to give permission for this activity.

For non-NHS sites, site management permission should be obtained in accordance with the procedures of the relevant host organisation.

Sponsors are not required to notify the Committee of management permissions from host organisations.

Registration of Clinical Trials

All clinical trials (defined as the first four categories on the IRAS filter page) must be registered on a publically accessible database. This should be before the first participant is recruited but no later than 6 weeks after recruitment of the first participant.

There is no requirement to separately notify the REC but you should do so at the earliest opportunity e.g. when submitting an amendment. We will audit the registration details as part of the annual progress reporting process.

To ensure transparency in research, we strongly recommend that all research is registered but for non-clinical trials this is not currently mandatory.

If a sponsor wishes to request a deferral for study registration within the required timeframe, they should contact hra.studyregistration@nhs.net. The expectation is that all clinical trials will be registered, however, in exceptional circumstances non registration may be permissible with prior agreement from the HRA. Guidance on where to register is provided on the HRA website.

It is the responsibility of the sponsor to ensure that all the conditions are complied with before the start of the study or its initiation at a particular site (as applicable).

Ethical review of research sites

The favourable opinion applies to all NHS sites taking part in the study, subject to management permission being obtained from the NHS/HSC R&D office prior to the start of the study (see "Conditions of the favourable opinion").

Extract of the meeting minutes

<u>Social or scientific value; scientific design and conduct of the study</u>

The PRS Sub-Committee commented that the answer to A62 of the IRAS Form, on the methods of analysis, was not explained in lay language and quite vague. The members were also unclear on how the study objectives would be achieved and serve the research question so agreed to ask the Applicant to clarify this, in lay language.

Dr McGilligan informed the Committee that exploratory data analysis techniques would be used to investigate relationships between variables in the database.

One such analysis to be used is a technique called association rule mining. The ultimate goal is to identify associations among uncategorised data. For example, such an analysis may show associations in a large dataset between gender, age and other risk factors for heart attacks such as diabetes or importantly may uncover new associations that could be used to generate new hypotheses to be tested in the future.

Another data analysis method to be used is a time series analysis. This will involve looking at the number of people with different characteristics or attributes (e.g. age, gender, and other comorbidities like diabetes) that have been referred on to the primary percutaneous intervention (pPCI) pathway overtime. This analysis will use this data to help forecast or predict what patients (with similar characteristics/attributes) that might be referred in the future.

Data mining techniques such as supervised and unsupervised clustering will also be used for discovering patterns in the data with an overall goal to extract information (with intelligent methods) from a data set and transform the information into a comprehensible structure for further use. The machine will use a large dataset with known outcomes for training itself. So the machine will learn rules e.g. people of a certain age and gender and with diabetes are more likely to have a heart attack and referred on to the pPCI pathway. A test dataset (data previously unseen by the data-mining algorithms) is then used to validate these rules. This is achieved by predicting which patients are more likely to have a heart attack and referred on to the pPCI pathway based on their individual attributes/characteristics that were used to inform the rules.

A final type of supervised learning-based analysis to be used is a decision tree machine learning algorithm. This produces a flowchart-like structure in which each internal node represents a "test" on an attribute, each branch represents the outcome of the test, and each leaf node represents a class label (decision taken after computing all attributes). The paths from root to leaf represent classification rules.

Dr McGilligan explained that all of these different techniques would allow them to retrospectively analyse the pPCI dataset and train the model for the prediction of similar types of data. This predictive model could potentially in the future, help nurses in their clinical decision making, which is the primary objective of the study.

The PRS Sub-Committee was satisfied with the responses detailed above.

<u>Care and protection of research participants; respect for potential and enrolled participants' welfare and dignity</u>

The PRS Sub-Committee agreed to seek confirmation that data would be completely anonymised before it was used for research and given to the researchers.

Dr McGilligan confirmed that the data would be completely anonymised before it would be used for research and given to researchers.

The PRS Sub-Committee was unclear whether or not the study would be purely looking at anonymised retrospective data and agreed to seek clarification from the Chief Investigator.

Dr McGilligan reassured the Committee that the project would only be looking at anonymised retrospective data.

The PRS Sub-Committee was satisfied with the responses detailed above.

Approved documents

The documents reviewed and approved were:

Document	*Version*	*Date*
Covering letter on headed paper [cover letter]		29 November 2018
Evidence of Sponsor insurance or indemnity (non NHS Sponsors only) [sponsor letter]		05 December 2018
IRAS Application Form [IRAS_Form_10122018]		10 December 2018
Letter from funder [letter from funder]		24 January 2017
Letter from sponsor [sponsorship form]		05 December 2018
Other [UU Ethics Filter Committee Report]		21 November 2018
Other [Student CV]		
Other [Supervisor CV]		
Other [Supervisor CV]		22 October 2018
Referee's report or other scientific critique report [RG2]		23 October 2018
Research protocol or project proposal [research protocol]	1.0	27 September 2018
Summary CV for Chief Investigator (CI) [cv]		22 October 2018
Summary CV for student [cv]		
Summary CV for supervisor (student research) [cv]		

Membership of the Proportionate Review Sub-Committee

The members of the Sub-Committee who took part in the review are listed on the attached sheet.

Statement of compliance

The Committee is constituted in accordance with the Governance Arrangements for Research

Ethics Committees and complies fully with the Standard Operating Procedures for Research Ethics Committees in the UK.

After ethical review

Reporting requirements

The attached document "After ethical review – guidance for researchers" gives detailed guidance on reporting requirements for studies with a favourable opinion, including:

- Notifying substantial amendments
- Adding new sites and investigators
- Notification of serious breaches of the protocol
- Progress and safety reports
- Notifying the end of the study

The HRA website also provides guidance on these topics, which is updated in the light of changes in reporting requirements or procedures.

User Feedback

The Health Research Authority is continually striving to provide a high quality service to all applicants and sponsors. You are invited to give your view of the service you have received and the application procedure. If you wish to make your views known please use the feedback form available on the HRA website:
http://www.hra.nhs.uk/about-the-hra/governance/quality-assurance/

HRA Training

We are pleased to welcome researchers and R&D staff at our training days – see details at
http://www.hra.nhs.uk/hra-training/

With the Committee's best wishes for the success of this project.

**18/LO/2203**	**Please quote this number on all correspondence**

Yours sincerely

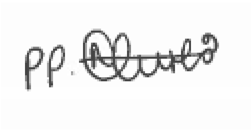

Dr Simon Walton
Chair

Email: NRESCommittee.SECoast-BrightonandSussex@nhs.net

Enclosures: *List of names and professions of members who took part in the review*

"After ethical review – guidance for researchers"

Copy to: *Mr Nick Curry*
Mrs Mary McGonigle, WHSCT, CTRIC
Lead Nation
Northern Ireland: research.gateway@hscni.net

London - Brighton & Sussex Research Ethics Committee

Attendance at PRS Sub-Committee of the REC meeting in correspondence

Committee Members:

Name	*Profession*	*Present*	*Notes*
Dr Caroline Garrett	Human Tissue Governance Manager and Research Fellow	Yes	
Dr Nicola Mason	Consultant Midwife	Yes	
Dr Simon Walton	Consultant in Anaesthesia and Intensive Care	Yes	(Chair)

Also in attendance:

Name	*Position (or reason for attending)*
Miss Nicole Curtis	REC Manager
